# Identifying ‘avoidable harm’ in family practice: a RAND/UCLA Appropriateness Method consensus study

**DOI:** 10.1186/s12875-019-0990-z

**Published:** 2019-10-04

**Authors:** Andrew Carson-Stevens, Stephen Campbell, Brian G. Bell, Alison Cooper, Sarah Armstrong, Darren Ashcroft, Matthew Boyd, Huw Prosser Evans, Rajnikant Mehta, Christina Sheehan, Aziz Sheikh, Anthony Avery

**Affiliations:** 1 Division of Population Medicine, School of Medicine, Cardiff University, 8th Floor Neuadd Meirionnydd, Cardiff, UK; 2 Australian Institute of Health Innovation, Macquarie University, Sydney, Australia; 3 Division of Population Health, Health Services Research & Primary Care, University of Manchester, Manchester, UK; 4 School of Medicine, University of Nottingham, Nottingham, UK; 5 Drug Usage and Pharmacy Practice Group, University of Manchester, Manchester, UK; 6 School of Pharmacy, University of Nottingham, Nottingham, UK; 7 Usher Institute of Population Health Sciences and Informatics, University of Edinburgh, Edinburgh, UK; 8 Harvard Medical School, Harvard University, Boston, USA

**Keywords:** Patient safety, adverse event, harm, Primary care, Family practice

## Abstract

**Background:**

Health care-related harm is an internationally recognized threat to public health. The United Kingdom’s national health services demonstrate that upwards of 90% of health care encounters can be delivered in ambulatory settings. Other countries are transitioning to more family practice-based health care systems, and efforts to understand avoidable harm in these settings is needed.

**Methods:**

We developed 100 scenarios reflecting a range of diseases and informed by the World Health Organization definition of ‘significant harm’. Scenarios included different types of patient safety incidents occurring by commission and omission, demonstrated variation in timeliness of intervention, and conditions where evidence-based guidelines are available or absent. We conducted a two-round RAND / UCLA Appropriateness Method consensus study with a panel of family practitioners in England to define “avoidable harm” within family practice. Panelists rated their perceptions of avoidability for each scenario. We ran a k-means cluster analysis of avoidability ratings.

**Results:**

Panelists reached consensus for 95 out of 100 scenarios. The panel agreed avoidable harm occurs when a patient safety incident could have been probably, or totally, avoided by the timely intervention of a health care professional in family practice (e.g. investigations, treatment) and / or an administrative process (e.g. referrals, alerts in electronic health records, procedures for following up results) in accordance with accepted evidence-based practice and clinical governance. Fifty-four scenarios were deemed avoidable, whilst 31 scenarios were rated unavoidable and reflected outcomes deemed inevitable regardless of family practice intervention. Scenarios with low avoidability ratings (1 s or 2 s) were not represented by the categories that were used to generate scenarios, whereas scenarios with high avoidability ratings (7 s 8 s or 9 s) were represented by these a priori categories.

**Discussion:**

The findings from this RAND/UCLA Appropriateness Method study define the characteristics and conditions that can be used to standardize measurement of outcomes for primary care patient safety.

**Conclusion:**

We have developed a definition of avoidable harm that has potential for researchers and practitioners to apply across primary care settings, and bolster international efforts to design interventions to target avoidable patient safety incidents that cause the most significant harm to patients.

**Electronic supplementary material:**

The online version of this article (10.1186/s12875-019-0990-z) contains supplementary material, which is available to authorized users.

## Introduction

Health care-related harm is an internationally recognized threat to public health and wellbeing. There is a global transition towards primary care-led health care systems [1] and countries like the United Kingdom demonstrate upwards of 90% of care encounters can be delivered in ambulatory settings [2]. As other countries transition to emulate those with predominantly extended family practice-based care models, a clear understanding of avoidable harm is needed to enable health care systems to identify and learn from the most serious incidents and the contributory factors amenable to intervention.

Most patient safety research has focused on specialist-care settings resulting in a greater awareness of the frequency and causes of health care-related errors, and the resulting burden of disease [3–5]. Patient safety research in primary care has been slower [1, 5] although the profile of patient safety in primary care is increasing through the World Health Organization’s (WHO) Safer Primary Care Expert Group, and more recently by the US National Patient Safety Foundation’s call to look “beyond hospitals to the full care continuum” [5–8]. A recent systematic review investigating the frequency and burden of harm in family practice concluded 2–3% of primary care encounters involve a health care-related error, and around one in 25 of those result in a significant harm outcome that has a substantial impact on a patients’ well-being. Included studies were notably heterogeneous in terms of their variability in study design and inconsistent definitions of outcome measures [9].

The WHO has recognized that standardized definitions of core terminology needs to be developed to permit the identification of health care-related harms in primary care and comparisons across settings, countries and over time [9]. We aimed to define “avoidable harm” to be used in future observational studies in family practice.

## Methods

### RAND/UCLA Appropriateness Method

The RAND/UCLA Appropriateness Method, an established approach for the development of health indicators [10–14], was used to develop a definition of “avoidable harm” to be understood and applied in family practice contexts. This method is used to combine scientific evidence with the collective judgement of experts (e.g. practising FPs) to achieve a consensus opinion from the group [10]. For example, experts are typically provided with hypothetical scenarios and an overview of relevant research evidence to support their decision-making, which in this case will be about the “avoidability” of the incidents that led to a significant harm outcome (this process is described in more detail later) [11–14].

### Generation of scenarios

Scenarios were developed by members of the research team (ACS, AC, HPE, AA) with extensive experience in analyzing patient safety incident reports from family practice [15]. Our working definition of “significant harm” was informed by an international classification of patient safety developed by the WHO and was inclusive of definitions of moderate harm, severe harm and death outcomes (see Table 1) [16]. This meant that we focused on harm outcomes that have more than a temporary impact on patients (i.e., extra observation, investigation, review or minor treatment).

**Table 1:** Working definition of “significant harm” in primary care

A patient harm outcome is symptomatic with one or all of the following: required more intensive intervention than might otherwise have been required (e.g., additional operative procedure; additional therapeutic treatment); resulted in an escalation of care (e.g., hospital admission, more urgent review in a secondary or tertiary care setting); caused a loss of function of at least one bodily organ, which may have been temporary or permanent; and, death.	

We identified 20 significant harm examples to reflect a diverse range of International Classification of Diseases 10th Edition (ICD-10) categories, each with 5 different scenarios (100 scenarios in total, see Additional file 1 for examples). A matrix was used to guide the scenario writing process and we endeavoured to include the following characteristics: a range of unavoidable to avoidable conditions; different types of patient safety incidents (e.g. medication errors, communication failures); errors of commission (i.e., doing something wrong) and omission (i.e., failing to do the right thing). The scenarios were amended and finalized following discussion with a RAND/UCLA Appropriateness Method expert (SC), the research team and a pilot exercise with practising FPs.

### Research evidence for each scenario

Relevant and current best-evidence guidelines (e.g. National Institute for Health and Care Excellence [NICE] and Scottish Intercollegiate Guidelines Network [SIGN] guidelines) were identified for each scenario. These were compiled in a supplementary research evidence document that experts were advised to consult for each scenario. If an evidence base for the scenario was not available, the experts were requested to apply the Bolam test [17, 18]; that is, to apply the standards they believe would be held by a responsible body of medical opinion.

### Recruitment of panelists

We recruited FPs through contacts at the Royal College of General Practitioners’ faculties in the East Midlands, London and the North-West of England. Contacts distributed an invitation email to FPs to participate in the consensus building exercise. FPs were eligible to take part in the study if they have had at least 5 years’ experience clinical practice. FPs were excluded if they had been barred from practising by the GMC.

### Two-round consensus process

In round one (October 2015), the “panel members” (i.e., experts) were invited to complete an online survey that contained the 100 scenarios (included in Additional file 1). They were required to read each scenario and the relevant accompanying evidence. Panelists were provided with our working definition of avoidability, developed within the research team, which was “an error of omission (failing to do the right thing) or commission (doing something wrong) in health care management that reflects a failure to follow acceptable practice at an individual or system level”. They were then asked to use their professional experience as a practitioner, in conjunction with the evidence summaries provided, to judge the extent the scenarios were avoidable using a 9-point Likert-type scale that ranged from 1 = “totally unavoidable” to 9 = “totally avoidable” (see Table 2 for definitions and examples).

**Table 2:** Frequency of categories of avoidability with definitions and examples

Rating	Category of avoidability	No. scenarios	Definition	Examples
1	Totally unavoidable	*n* = 18	Family Practice professionals have adhered to all appropriate evidence-based guidelines or best practice at the level of the health care professional (e.g. knowledge or skill errors) and / or the practice (e.g. administrative processes). There is no absolute causation between the identified error(s) and the harm outcome. Family practice could have done no more.	A patient on methotrexate receives evidence-based blood test monitoring. An urgent blood test is undertaken when there is a suspicion about immune-compromise underlying a presentation with a chest infection. Patient is advised to withhold methotrexate whilst taking antibiotics. Despite the FPs efforts to treat the infection, and worsening advice provided, the patient is admitted to hospital following deterioration with sepsis. A 55-year old man presented with an acute MI. He was an asylum seeker and had not as yet registered with a FP.
2–3	Probably unavoidable	*n* = 13	Family Practice professionals have adhered to appropriate evidence-based guidelines or best practice at the level of the health care professional (e.g. knowledge or skill errors) and / or the practice (e.g. administrative processes). There is considerable doubt concerning absolute causation between the identified error(s) and the harm outcome and if family practice could have done more.	A patient attends with a tonsillar lesion and is prescribed antibiotics and advised to return in 2 weeks for review. The patient did not return for 4 weeks, at which point the FP deemed an urgent referral to ENT was indicated and an inoperable squamous cell carcinoma was diagnosed. A patient with a 12-month history of atrial fibrillation presents with an ischemic stroke. The patient has been prescribed anticoagulation, and INR has been in the target range for 90% of the time over the previous 6 months.
4–6	Possibly avoidable	*n* = 10	Family Practice professionals have adhered to all appropriate evidence-based guidelines and / or the practice (e.g. administrative processes). There is some doubt concerning absolute causation between the identified error(s) and the harm outcome. It is unclear whether the event would have been avoidable with more input from family practice.	A patient with diabetes is the main carer for his wife with dementia. He has declined a specialist referral for retinal screening in the past for this reason. He presents with sudden onset blindness due to a retinal detachment on a background of diabetic retinopathy. A patient is admitted to hospital with an upper gastrointestinal bleed. The patient is 45 years old with a history of coronary heart disease on regular aspirin therapy.
7–8	Probably avoidable	*n* = 53	‘Probably avoidable’ was judged by the same criterion as possibly avoidable although there is less doubt concerning absolute causation between the identified error(s) and the harm. The outcome may not have occurred with more family practice intervention.	A patient on methotrexate that has not had the recommended blood monitoring is admitted to hospital with a pneumonia and a low blood white cell count. A patient aged 50 is diagnosed with Bladder Cancer. The patient had presented with symptoms of frequency and pain and despite not having any bacterial growth on several MSUs, had red blood cells 5–99 on each one. The FP seeing her did not realize the relevance of this finding and referred her as a routine referral and not less than 2-week wait. She had metastatic disease after an 18 week wait.
9	Totally avoidable	*n* = 1	Outcome experienced by the patient is directly attributed to the demonstrable failure in family practice to adhere to evidence-based or best practice at the level of the health care professional (e.g. knowledge or skill errors) and / or the practice There is a clear and absolute causation between the identified error(s) and the harm outcome. The harm would not have occurred with more family practice intervention. Family practice should have done more.	Prescribing a non-steroidal anti-inflammatory drug (NSAID) to a patient also taking Warfarin which resulted in an upper gastrointestinal bleed.

All data were exported to a Microsoft Excel (Redmond, Washington: Microsoft, 2010) spreadsheet and the median score for all items and the percentage agreement for items scoring “7 and 8” (“probably avoidable”) or 9 (“totally avoidable”) i.e., the frequency of the highest scores were calculated. The medians and percentage agreements obtained for each item were then included in the revised survey that formed the basis for round two of data collection, giving participants the opportunity to revise their scoring on the basis of other participants’ rankings.

In round two, panel members met for a one-day face-to-face meeting (November 2015), co-chaired by a RAND/UCLA Appropriateness Method expert (SC) and an experienced FP (AC). Field notes were made by two non-participant observers (BB and CS) and a medically qualified researcher with qualitative expertise (ACS). The panel discussed each scenario as a group, and following those discussions independently re-rated each scenario. Each panelist had a customized printed rating sheet that included their initial round one rating, and for comparison, the frequency distribution of ratings from all other panelists (anonymized) and the overall group median rating.

During round two, panelists rated the scenarios as written. All panelists participated in discussions, and those with outlying scores for a scenario had the opportunity to explain their justifications. In addition, wider justification was sought from the group around why they had reached consensus for each scenario. It enabled an exploration of areas of convergence and divergence across scenarios, giving participants the opportunity to identify the actions, conditions and characteristics of “avoidable harm”. These discussions enabled an iterative development of the definitions for each avoidability category.

### Analysis

During round two, the level of consensus within the panel for each scale for each scenario was calculated in real-time. Ratings were: ‘unavoidable’ if the overall panel median ratings were in the tertile 1–3; ‘possibly avoidable’ if the overall panel median ratings were in the tertile 4–6; and, ‘avoidable’ if the overall panel median ratings were in the tertile 7–9. Agreement signified that no more than 20% of panelists’ ratings were outside the same 3-point tertile (that is, 1–3, 2–4, 4–6, 7–9) as the observed median (i.e., for a 12 person-panel, no more than 2 ratings outside any 3-point tertile). This method was identical to the one used in our previously published research [12]. Results are presented for the final (round two) ratings only. Observational field notes taken during panel discussions by ACS, CS and BB support our interpretation of the study findings [19].

We ran a post-hoc k-means cluster analysis on the avoidability ratings of the 100 scenarios using a 6-cluster solution and updated cluster centres iteratively. The 6-cluster solution was chosen because this was the number of characteristics (e.g. errors of omission or commission, timeliness issues) that were used by the team to develop the scenarios originally. We then classified the items in each cluster according to the avoidability rating of that cluster as well as which of the 6 characteristics that were used to develop the scenarios that cluster represented.

## Results

### Summary of participants

Twelve FPs participated as panelists from East Midlands (*n* = 9 (75%)) and London (*n* = 3 (25%)), England, with a roughly equal mix of males (*n* = 5 (42%)) and females (*n* = 7 (58%)). All participants were FPs with a specialist or generalist knowledge of patient safety. All 12 panelists participated in both rounds and were remunerated (£600 each) for their participation.

Panelists reached consensus for 95 scenarios (95%). Only five scenarios (5%) lacked consensus and this was due to differences in opinion of whether the harm arose from primary or secondary care.

### Consensus categories of avoidability

Definitions of each avoidability category were iteratively developed by the panelists during round 2 discussions (Table 2).

If the outcome was directly attributable to the event described, then it was defined as *totally avoidable* (*n* = 1, rating of 9); for example, in scenario B5, where a patient was admitted to hospital with a gastrointestinal bleed following concurrent prescription of *warfarin in combination with an oral non-steroidal anti-inflammatory drug over the previous two months.* However, if there was any doubt that the event was directly attributable (there was a ‘but’) it became a 7 or 8 (*n* = 53, *probably avoidable*). For example, in scenario F5: a 60-year-old patient on methotrexate for rheumatoid arthritis did not have white cell count monitoring for six months. The patient presented to the family practice with an infection that was treated with antibiotics although later deteriorated and was admitted to hospital where a low white cell count was identified.

If the panelists felt that attribution was 50/50 or the scenario did not give them enough information to decide either way, either a rating of 4, 5 or 6 was given (*n* = 10, *possibly avoidable*). For example, in scenario C3, a patient was diagnosed with a malignant melanoma. He had attended the practice two years previously with a pigmented lesion at the same site as the melanoma with a note in the records stating: ‘Pigmented lesion left forearm. o/e: pigmented lesion - no evidence of malignancy’.

Alternatively, if the outcome was not felt to be directly attributable to the event described at all, then it was defined as *totally unavoidable* (*n* = 18, rating of 1); for example, in scenario J1 where the patient experienced a ruptured ectopic pregnancy: “The patient was seen that day by the [FP] and assessed, a pregnancy test was positive and she was admitted immediately.” If there was any doubt that the event was not directly attributable (i.e., there was a ‘but’) it became a 2 or 3 (*n* = 13, *probably unavoidable)*. For example, in scenario E4 whilst the patient had an inoperable tonsillar squamous cell carcinoma, a possible suspicious lesion was noted on the left tonsil and was treated with antibiotics with the advice follow up was required one week later to finalise a decision about referral.

### Cluster analysis of avoidability ratings

The cluster analysis revealed 29 scenarios were in the first cluster, 6 were in the second cluster, 6 were in the third cluster, 54 were in the fourth cluster, 4 were in the fifth cluster, and 1 was in the sixth cluster.

Scenarios in cluster 1 tended to not fall in at least one of the 6 characteristics (26 out of 29 scenarios did not fall into any of the 6 characteristics) and these scenarios were also given avoidability ratings of either 1 or 2. In contrast, for the scenarios in cluster 4, all of the scenarios fell into one or more of the 6 categories with most scenarios represented by the following characteristics: omission (32 out of 54), timeliness of intervention (44 out of 54) or ‘not evidence based’ (40 out of 54). All of these scenarios were given avoidability ratings of 7 or 8.

We also classified the scenarios in cluster 2, 3, 5, and 6 in the same way and, with the exception of cluster 6 that contained only one item, the scenarios in all of the clusters tended to receive equivocal avoidability ratings and/or consensus could not be reached with respect to avoidability.

## Discussion

### Main findings

From our analysis of avoidable harm scenarios, agreed by FPs participating in a modified RAND/UCLA Appropriateness Method process, we have derived a definition of avoidable harm in the context of family practice:

“a patient safety incident could have probably, or totally been avoided by the timely intervention of a health care professional in family practice (e.g. investigations, treatment, safety netting) and / or an administrative process (e.g. referrals, alerts in electronic health records, procedures for following up results) in accordance with accepted standards of evidence-based practice and / or clinical governance and / or the Bolam test [17, 18].”

Scenarios with low avoidability ratings (1 s or 2 s) were not represented by the characteristics included in the above definition, whereas scenarios with high avoidability ratings (7 s 8 s or 9 s) were represented by these characteristics.

### Discussion of findings in relation to existing literature

Primary care patient safety is an emerging international policy agenda, and this is signaled by the release of the WHO’s Technical Series for Safer Primary Care where world experts have explored the existing evidence base for primary care safety [16]. Multiple systematic reviews and professional reports have highlighted major evidence gaps exist and more high-quality epidemiological studies are needed [2–6, 8, 20]. Clinical case note review has been the method of choice that has informed the extensive knowledge and understanding generated about healthcare-associated harm in hospital settings [8]. Previous systematic reviews of studies to estimate the frequency and burden of unsafe primary care have demonstrated considerable variation in the quality of included studies, and retrospective methods yield lower estimates, than those generated by prospective observations [9].

A wide range of classification systems have been used with differing definitions of harm severity [21]. This presents a challenge when making comparisons. In recent years, the WHO developed the International Classification for Patient Safety to standardize the concepts and terminology used in the field [16]. This study builds on the work undertaken already by WHO by defining “avoidability” within the context of family practice and begins to frame the scope of inquiry that clinicians, researchers and policymakers must now endeavor to understand.

### Implications for practice, policy, or future research

Understanding the epidemiology of errors in family practice contexts is crucial for establishing a baseline, identifying areas of practice most amenable to improvement, and the development of interventions to reduce the risk of healthcare-associated harm to patients [9]. Many countries, particularly with low and middle income economies, are seeking to develop predominantly family practice-based care models. It is important and timely to understand the significant, avoidable healthcare-related sources of harm arising in these settings and at scale.

The findings from this RAND/UCLA Appropriateness Method study define the characteristics and conditions that can be used to standardize research processes for measurement of outcomes for primary care patient safety. Whilst there are often explicit criteria that sensitize reviewers to well-known risk factors for harm that trigger more in-depth review, the nature of error in family practice – as demonstrated by our definition – means that such criteria could be challenging for application. Thus, review processes that utilize the expert judgement and tacit knowledge of clinical reviewers must be embraced. This consensus study has set the boundaries and established the conditions for this implicit process of inquiry.

### Strengths and limitations of the study

Our study was strengthened by our matrix created for the refinement and testing of candidate scenarios. This was developed by members of our team of whom have completed the largest evaluation of family practice patient safety incidents reports and the most current systematic reviews of primary care safety [4, 6, 15]. Our approach provided a large, diverse number of scenarios (*n* = 100). Whilst we do not claim the scenarios represent all possible unsafe incidents in primary care, they do represent the most common and most severe incidents identified from our program of family practice research in the National Health Service (NHS) [4, 9, 15, 22–26], and efforts led by others [27–31]. The NHS is a publicly funded, single-payer service and those practicing in other systems should interpret the transferability of our definition cautiously by considering relevant amendments for their own context. Our pilot definition of “avoidable harm” will now be tested in a large, comprehensive, case note review study in family practices in England.

This study adhered to a validated systematic consensus method to identify the level of avoidability represented by each scenario [9]. Panelists were recruited via the RCGP network in three of 32 faculties chosen to recruit GP from regions with a mix of rural, urban and inner-city practices. Panelists experienced difficulty judging whether the health care professional in family practice could have done more to intervene or act in a timelier manner (e.g. called the hospital rather than sent a further expedite letter), particularly when they were otherwise following what would be deemed to be evidence-based practice. We accept there will be variation in guideline preferences between countries, and acknowledge the actions taken by FPs in our scenarios may not be possible in some contexts. In some scenarios, the health care professional in family practice needed more information to make a decision and our experienced panelists could appreciate the importance of watchful waiting. This highlights the potential value of encouraging professional discussions about avoidability. In practice, we would strongly advocate cases of avoidable harm are discussed in the spirit of identifying systemic weaknesses compromising the ability of FPs and the wider primary care team to deliver safe care.

## Conclusion

Our definition of “avoidable harm” has potential to support researchers and practitioners to clarify the scope of inquiry needed to determine the frequency and burden of unsafe family practice. This could enable international comparison of findings that should accelerate the pace of learning to design and implement interventions to improve patient safety across a range of FP contexts and economic circumstances.

## Additional file

Additional file 1: Avoidability ratings from Round 2 of RAND / UCLA consensus process. (DOCX 36 kb)

## Data Availability

All raw study data can be obtained upon request from the corresponding author.

## Abbreviations

FP: Family practitioner
ICD-10: International Classification for Disease version 10
IRB: Institutional Review Board
NHS: National Health Service
RAND: Research and Development
UCLA: University of California at Los Angeles
WHO: World Health Organization
GMC: General Medical Council
