# Supplementary material for: Identifying ‘avoidable harm’ in family practice: a RAND/UCLA Appropriateness Method consensus study
Source: BMC Fam Pract. 2019 Oct 4;20:134. doi: 10.1186/s12875-019-0990-z (PMC6777037; doi:10.1186/s12875-019-0990-z)
Supplement: Supplementary file 1 — Avoidability ratings from Round 2 of RAND / UCLA consensus process. (DOCX 36 kb) [file 12875_2019_990_MOESM1_ESM.docx]

**Additional file 1: Avoidability ratings from Round 2 of RAND / UCLA consensus process**

| **Significant harms (A-T)** with different scenarios (numbered) for assessing avoidability  Avoidable with consensus ***in bold italics*** | | **Median rating** | **Category of avoidability** |
| --- | --- | --- | --- |
| **A.** | **Patient with a 12-month history atrial fibrillation presents with an ischemic stroke** | | |
| 1 | Patient has been prescribed anticoagulation, and INR has been in the target range for 90% of the time over the previous 6 months. | 2 | Probably  Unavoidable |
| 2 | ***Patient has been prescribed anticoagulation, and INR has been in the target range for 50% of the time over the previous 6 months*** | ***7*** | ***Probably***  ***Avoidable*** |
| 3 | Patient had a CHA2DS2-VASc score of 1 and had been prescribed aspirin rather than an anticoagulant for prophylaxis of stroke. | 5 | Possibly  Avoidable |
| 4 | ***Patient had a CHA2DS2-VASc score of 3 and had been prescribed aspirin rather than an anticoagulant for prophylaxis of stroke.*** | ***8*** | ***Probably***  ***Avoidable*** |
| 5 | Patient had a CHA2DS2-VASc score of 3 and had been prescribed no medication for prophylaxis of stroke as the patient had declined | 2 | Probably  Unavoidable |
| **B.** | **Patient admitted to hospital with upper gastrointestinal bleed** | | |
| 1 | ***Patient aged ≥65 years had received two prescriptions for ibuprofen 400mg three times daily (quantity 84 each time) in the previous 2 months for musculoskeletal pain without co-prescription of an ulcer-healing drug*** | ***8*** | ***Probably***  ***Avoidable*** |
| 2 | Patient aged ≥65 years had received two prescriptions for ibuprofen 400mg three times daily (quantity 84 each time) in the previous 2 months for musculoskeletal pain with co-prescription of an ulcer-healing drug | 6 | Possibly  Avoidable |
| 3 | ***Patient aged 45 years had received two prescriptions for naproxen 500mg twice daily (quantity 60 each time) in the previous 2 months for musculoskeletal pain, without co-prescription of an ulcer-healing drug.*** | ***7*** | ***Probably***  ***Avoidable*** |
| 4 | Patient aged 45 years with a history of coronary heart disease on regular aspirin therapy. | 5 | Possibly  Avoidable |
| 5 | ***Patient aged 75 years with atrial fibrillation and musculoskeletal pain had received a regular prescription of warfarin in combination with an oral NSAID over the* previous two months.** | ***9*** | ***Totally***  ***Avoidable*** |
| **C.** | **A Patient is diagnosed with malignant melanoma left forearm. He had attended the surgery 2 years previously with a pigmented lesion at the same site as the melanoma with a note in the records stating:** | | |
| 1 | ***‘Pigmented lesion left forearm. Patient thinks this may have increased in size over recent months. No itching or bleeding. o/e: pigmented lesion approx. 1cm in diameter, slightly irregular edge and irregular color. Refer 2 week wait’. No referral took place.*** | ***8*** | ***Probably***  ***Avoidable*** |
| 2 | ***‘Pigmented lesion left forearm. Patient thinks this may have increased in size over recent months. No itching or bleeding. o/e: pigmented lesion approx. 1cm in diameter, slightly irregular edge and irregular color. Impression: probably benign. Patient advised to return if further changes in size or shape. Patient never represented.*** | ***8*** | ***Probably***  ***Avoidable*** |
| 3 | ‘Pigmented lesion left forearm. o/e: pigmented lesion - no evidence of malignancy’ | 5 | Possibly  Avoidable |
| 4 | ‘Pigmented lesion left forearm o/e 1cm diameter, regular edge and pigmentation. Reassured looks benign but advised if any change to see dr.’ Patient never represented. | 2 | Probably  Unavoidable |
| 5 | ***‘hand pain – typical osteoarthritic changes base, gen advice and analgesia. Blood pressure 130\80 well controlled on ACE inhibitor, recent U&Es normal, continue. Bowel symptoms improved, no weight loss, normal exam and FBC last time, advised if reoccurs to see doctor. Pigmented lesion left forearm, some irregularity of border and pigmentation but looks benign, see doctor if changes.’ Patient never represented.*** | ***7*** | ***Probably***  ***Avoidable*** |
| **D.** | **A 16-year-old girl was admitted acutely requiring an emergency laparotomy for Crohn’s disease having previously presented to the GP with a 6-week history of bloody diarrhea and weight loss.** | | |
| 1 | ***The GP referred her urgently to gastroenterology. An expediting letter was sent 2 months later as she still had not been seen.*** | ***7*** | ***Probably***  ***Avoidable*** |
| 2 | ***An urgent gastroenterology referral was planned but never sent as the GP then became involved in an emergency. A referral was sent 2 months later when she represented.*** | ***8*** | ***Probably***  ***Avoidable*** |
| 3 | ***An urgent gastroenterology referral was done and she was seen within the month and treatment commenced. The specialist sent a letter to the GP advising treatment and a prescription was done but for the wrong drug dose.*** | ***7*** | ***Probably***  ***Avoidable*** |
| 4 | An urgent gastroenterology referral was done and she was seen within the month and treatment planned. The specialist sent a letter to the GP advising oral steroid therapy but the letter was delayed and therefore treatment was delayed for 2 weeks. | 3 | Probably  Unavoidable |
| 5 | ***The patient spoke with the triage nurse, was reassured and no appointment was offered.*** | ***8*** | ***Probably***  ***Avoidable*** |
| **E.** | **A 55-year-old man is diagnosed with an inoperable tonsillar squamous cell carcinoma** | | |
| 1 | He was noted to have a lesion on his left tonsil and was referred under the 2-week rule to ENT. | 1 | Totally  Unavoidable |
| 2 | He presented with recurrent sore throats and was seen and examined 5 times before being referred under the 2-week rule to ENT. | 5 | Possibly  Avoidable |
| 3 | ***He rang the surgery to request antibiotics for a sore throat on 3 separate occasions and received amoxicillin. Two months later he was seen and referred under the 2-week rule to ENT.*** | ***8*** | ***Probably***  ***Avoidable*** |
| 4 | He was noted to have a lesion on his left tonsil and was treated with antibiotics but advised to be seen again the following week for review. He was then referred under the 2-week rule to ENT | 2 | Probably  Unavoidable |
| 5 | He was noted to have a lesion on his left tonsil and was treated with antibiotics but advised to be seen again the following week for review. He did not return for another 4 weeks. He was then referred under the 2-week rule to ENT | 2 | Probably  Unavoidable |
| **F.** | **A 60-year-old lady on methotrexate for rheumatoid arthritis, prescribed by the GP under a shared care agreement, presented with a lower respiratory tract infection and was commenced on antibiotics. She deteriorated and was admitted a week later.** | | |
| 1 | She attended regularly for blood monitoring and was advised to withhold her methotrexate while on antibiotics. Her FBC was checked which showed a normal WCC prior to admission | 1 | Totally  Unavoidable |
| 2 | ***She attended regularly for blood monitoring but was not advised to withhold her methotrexate while on antibiotics and an FBC was not checked. Her WCC was low on admission.*** | ***7*** | ***Probably***  ***Avoidable*** |
| 3 | She attended regularly for blood monitoring. She was not advised to withhold her methotrexate while on antibiotics but an FBC was checked that was normal. | 6 | Possibly  Avoidable |
| 4 | ***She attended regularly for blood monitoring. She was not advised to withhold her methotrexate while on antibiotics but an FBC was checked that showed a low WCC. The doctor marked the result as ‘see doctor’, the patient made an appointment for the following week but continued on her methotrexate in the meantime.*** | ***7*** | ***Probably***  ***Avoidable*** |
| 5 | ***Her last FBC was 6 months previously and her WCC was low on admission.*** | ***8*** | ***Probably***  ***Avoidable*** |
| **G.** | **A patient presents with sudden onset blindness in his left eye due to retinal detachment on a background of proliferative diabetic retinopathy.** |  |  |
| 1 | He has always been poorly compliant with his insulin therapy despite diabetic specialist nurse input. His average Hba1c is 100. He is a heavy smoker, and despite regular invitations, does not attend the diabetic clinic and retinopathy screening | 2 | Probably  Unavoidable |
| 2 | ***Despite taking metformin for many years, he was not on the practice diabetic register and therefore retinopathy screening invites were not sent.*** | ***8*** | ***Probably***  ***Avoidable*** |
| 3 | The patient was under specialist care for diabetes and known retinopathy | 1 | Totally  Unavoidable |
| 4 | ***He presented with symptoms of flashes and floaters a week previously to the triage nurse and was advised to make a routine optician appointment.*** | ***8*** | ***Probably***  ***Avoidable*** |
| 5 | The patient cares for his wife that suffers with dementia and has difficulty attending medical appointments. He has previously declined referral to secondary care for management of his diabetes for this reason. | 6 | Possibly  Avoidable |
| **H.** | **A 33/40 pregnant woman is admitted with preeclampsia which led to a pre-term delivery, having been seen by the GP 1 week previously complaining of a headache.** | | |
| 1 | ***Her blood pressure was not measured.*** | ***8*** | ***Probably***  ***Avoidable*** |
| 2 | ***Her blood pressure was 150/100 but not acted upon.*** | ***8*** | ***Probably***  ***Avoidable*** |
| 3 | ***Her blood pressure was 150/100 and the GP dictated an urgent referral to the obstetrician but this was never typed since the tape was lost. She did not represent until the day of the admission.*** | ***8*** | ***Probably***  ***Avoidable*** |
| 4 | ***Her blood pressure was 150/100 and the GP dictated an urgent referral to the obstetrician but this was never typed since the tape was lost.*** | ***8*** | ***Probably***  ***Avoidable*** |
| 5 | ***Her blood pressure was 140/100 and she was noted to be upset due to family problems that the GP spent time discussing with her. It was advised that her BP was repeated to following week.*** | ***7*** | ***Probably***  ***Avoidable*** |
| **I.** | **A 55-year old man presented with an acute MI.** | | |
| 1 | He was receiving treatment for hypertension. Last recorded BP 138/84 3 months ago. Lipids had not been checked. | 6 | Possibly  Avoidable |
| 2 | He was receiving treatment for hypertension and hypercholesterolemia. Last recorded BP 138/84 6 months ago, >40% reduction in non-HDL cholesterol. It appears he had stopped taking his medication for the previous 3 months. | 2 | Probably  Unavoidable |
| 3 | ***He was receiving treatment for hypertension. Last recorded BP 160/100 3 months ago. Lipids had not been checked.*** | ***7*** | ***Probably***  ***Avoidable*** |
| 4 | ***There was a single recorded BP of 160/100 2 years ago. Lipids had never been checked.*** | ***8*** | ***Probably***  ***Avoidable*** |
| 5 | He was an asylum seeker and had not as yet registered with a GP. | 1 | Totally  Unavoidable |
| **J.** | **An 18-year-old female was admitted with a ruptured ectopic pregnancy requiring urgent surgery. She had previously had contact with the GP regarding abdominal pain.** | | |
| 1 | The patient was seen that day by the GP and assessed, a pregnancy test was positive and she was admitted immediately. | 1 | Totally  Unavoidable |
| 2 | ***The GP conducted a full history and examination. Periods noted to be irregular but no pregnancy test done. She was given analgesia and advised to return if symptoms worsened.*** | ***8*** | ***Probably***  ***Avoidable*** |
| 3 | ***She spoke to the GP the previous week and was advised to take analgesia and return if symptoms worsened. No documentation that pregnancy had been discussed.*** | ***8*** | ***Probably***  ***Avoidable*** |
| 4 | ***She was admitted to the surgeons by the GP ?acute appendicitis, chance of pregnancy was not documented in the notes. The surgeons discharged her after normal blood tests.*** | ***7*** | ***Probably***  ***Avoidable*** |
| 5 | She was seen and assessed and denied any chance of pregnancy. She admitted that her IBS symptoms had also reoccurred and was given buscopan and advised to represent if symptoms worsened. | 3 | Possibly  Unavoidable |
| **K.** | **A 13-year-old boy required urgent surgery for a slipped upper femoral epiphysis. He had previously seen the GP regarding knee pain.** | | |
| 1 | ***The patient complained of knee pain for 4 weeks. The GP noted that gait and knee examination were normal and the patient was reassured. He presented again 4 weeks later with worsening pain. Knee examination was again normal but hip examination showed reduced internal rotation and he was referred urgently to orthopedics.*** | ***7*** | ***Probably***  ***Avoidable*** |
| 2 | ***The GP noted that gait and knee examination were normal but hip examination showed reduced internal rotation and he was referred urgently to orthopedics. The appointment was for 6 month’s time by which time he could not walk and was admitted acutely.*** | ***7*** | ***Probably***  ***Avoidable*** |
| 3 | The patient had a limp and reduced hip rotation. He was admitted acutely. | 1 | Totally  Unavoidable |
| 4 | ***The GP noted that gait and knee examination were normal but hip examination showed reduced internal rotation and he was referred urgently to orthopedics however the doctor went on an urgent house call and forgot to send the referral. The patient presented again 4 weeks later when he could not walk and was admitted acutely.*** | ***8*** | ***Probably***  ***Avoidable*** |
| 5 | ***The patient presented on 3 separate occasions with ongoing knee pain for 4 months. His mother suffered from anxiety and the family often attended. There was no documentation of hip examination. He presented again 4 weeks later with worsening pain and a limp and was admitted acutely.*** | ***8*** | ***Probably***  ***Avoidable*** |
| **L.** | **A patient’s renal function deteriorated after an ACEi was commenced for hypertension.** | | |
| 1 | The ACEi was stopped and an alternative antihypertensive was commenced. A repeat blood test confirmed that renal function had returned to normal. | 1 | Totally  Unavoidable |
| 2 | ***The GP marked the test result as ‘see doctor’ but the patient did not return for the results and continued the ACEi and resulted in CKD 4.*** | ***7*** | ***Probably***  ***Avoidable*** |
| 3 | ***The GP mistakenly marked the test result as ‘satisfactory’ so the patient continued the ACEi which resulted in CKD 4.*** | ***8*** | ***Probably***  ***Avoidable*** |
| 4 | ***The patient was not advised to have a U&E checked after treatment was commenced and was later admitted with an Acute Kidney Injury, renal function never returned to normal resulting in CKD 4.*** | ***8*** | ***Probably***  ***Avoidable*** |
| 5 | The patient had poorly controlled BP and was intolerant of many antihypertensives. The GP had discussed the need to monitor renal function and advised stopping the patient’s NSAIDS and furosemide prior to commencing the ACEi. Despite stopping the ACEi when renal function deterioration was noted 2 weeks later, the renal function never returned to normal resulting in CKD 4. | 2 | Probably  Unavoidable |
| **M.** | **A patient was diagnosed with metastatic breast carcinoma** | | |
| 1 | She had skin puckering in the lower half of her breast and didn’t notice it until examined by the GP due to an incidental finding of raised calcium and anemia. | 1 | Totally  Unavoidable |
| 2 | ***The patient had breast skin puckering and phoned the surgery for an appointment urgently. She was told she couldn’t have one for 4 weeks and decided to postpone as was frightened of outcome. She was seen 4 months later with pathological fracture.*** | ***8*** | ***Probably***  ***Avoidable*** |
| 3 | She was seen by a locum GP when she arranged an appointment to have her breast examined. She didn’t know the male doctor and was embarrassed so did not divulge the real reason for attending and asked for her BP to be checked. | 3 | Probably  Unavoidable |
| 4 | ***The patient saw the GP with a mass which appeared cystic. The GP had breast surgery experience and aspirated the cyst. Cytology was sent but the sample was mislabeled so not processed. The patient presented 4 months later with a pathological fracture.*** | ***8*** | ***Probably***  ***Avoidable*** |
| 5 | ***The patient sees the GP with an area of skin puckering; a referral is faxed to breast clinic. It is sent to the wrong number and no one picks this up. The patient calls back 8 weeks later and is then referred. She is found to have metastatic disease.*** | ***8*** | ***Probably***  ***Avoidable*** |
| **N.** | **A 4-year-old boy presents to hospital with DKA and requires a stay in intensive care before recovering** | | |
| 1 | He saw the GP for the first time with lethargy and dry looking lips with Kussmaul’s breathing. He has had a short illness which his parents have attributed to a viral illness which his little sister had the week before. GP takes a BM – notes ‘high’ and admits acutely. | 1 | Totally  Unavoidable |
| 2 | ***The mother calls the out of hours GP saying her son is unwell but gives a vague history. Nurse triage advised seeing own GP in the morning having used the algorithm for sore throat. As he had no temperature and mum couldn’t see if he had mottled skin and answered that his breathing was ‘ok, I guess’ the outcome from the algorithm was ‘see own GP’.*** | ***7*** | ***Probably***  ***Avoidable*** |
| 3 | ***The patient sees 4 GPs in the same practice over the course of 10 days, including 2 locum doctors, and once when computer systems were down, meaning they had no access to records. Each time felt to have a viral URTI. This is despite mum saying he was drinking more than usual and had lost weight and one GP noting “if not better next time - ?urinalysis?”. Eventually seen in out of hours when became floppy and lethargic. Admitted with unreadable BMs and Ketones.*** | ***8*** | ***Probably***  ***Avoidable*** |
| 4 | Patient’s father attempts to call GP surgery, asks for an appointment. Due to his limited English skills, he doesn’t understand the questions about whether it’s an emergency or not so hangs up in frustration. Boy is kept at home and given sweet tea for the night and when he is unrousable in the morning – an ambulance is called. | 5 | Possibly  Avoidable |
| 5 | ***A GP in out of hours sees the boy with a history of being generally unwell, wants to do a BM or urinalysis but none of the necessary equipment is available. GP decides to give the patient worsening advice and sends home. He is seen the next day by his own GP and is much worse – with high BMs and Ketones with Kussmaul breathing. Admission is arranged via 999 ambulance.*** | ***8*** | ***Probably***  ***Avoidable*** |
| **O.** | **A 25-year-old male who smokes large amounts of cannabis is admitted due to attempted suicide** | | |
| 1 | ***The patient is seen by a GP for an ingrowing toenail. Mentioned at the end of the consultation – he is feeling low. GP doesn’t carry out a risk assessment and asks him to return for a full assessment in another appointment. This is arranged for 3 weeks later. In the meantime, the low mood deteriorates and he attempts suicide 10 days later.*** | ***7*** | ***Probably***  ***Avoidable*** |
| 2 | The patient has never made contact with health care services and one night after failing his final exams, smokes a lot of cannabis, starts talking about ‘visions’ he is getting and runs away from his friends in his flat and jumps from a bridge. | 1 | Totally  Unavoidable |
| 3 | ***Patient is seen by his GP who knows him well. The GP recognizes his mental health has taken a turn for the worse. He is referred urgently via fax to CMHT. The referral is never received. The patient, never receives an assessment and no treatment changes are made. He attempts suicide the next week.*** | ***7*** | ***Probably***  ***Avoidable*** |
| 4 | Patient is referred to the CMHT with suicidal ideation. The crisis team are unable to see him and so arrange for his usual CPN to see him the next day, he is distraught by this and attempts suicide that night. | 1 | Totally  Unavoidable |
| 5 | ***The patient loses his anti-psychotic medication on a bank holiday weekend. There is a long wait to see an out of hours GP and in fact, once he speaks to a nurse on the telephone for triage, he is told to see his own GP on Tuesday. His hallucinations return and leads to him attempting suicide.*** | ***8*** | ***Probably***  ***Avoidable*** |
| **P.** | **Child of 11 is admitted to ITU with an exacerbation of asthma** | | |
| 1 | ***Child with asthma is having increasing exacerbations. GP fails to start Corticosteroid inhaler. Requires admission 3 days later.*** | ***7*** | ***Probably***  ***Avoidable*** |
| 2 | ***Child with asthma at stage 2 management with Ventolin and Clenil sees asthma nurse. Parents misunderstand management plan, and are not provided with a written plan. Child then takes Ventolin daily and Clenil prn. She suffers an exacerbation and requires ventilation*** | ***8*** | ***Probably***  ***Avoidable*** |
| 3 | A child with brittle asthma and on stage 4 management under secondary care has allergies to dogs and cats. Her mother refuses to get rid of the family pets. She experiences a life-threatening exacerbation despite being on best practice recommended management. | 1 | Totally  Unavoidable |
| 4 | ***A child with asthma does not attend for annual asthma review as the GP surgery has no automatic recall in place for these patients. It goes unnoticed that she has requested 6 Ventolin inhalers in 2 months and over a bank holiday weekend when she is unable to get a replacement inhaler – ends up needing A&E and ITU care.*** | ***8*** | ***Probably***  ***Avoidable*** |
| 5 | A child with no previous attendance at a health care facility with wheeze, attends A&E with wheeze, cyanosis, drowsiness and a silent chest. | 1 | Totally  Unavoidable |
| **Q.** | **A Woman of 60 has a PE** | | |
| 1 | ***She had been on combined continuous HRT for 15 years. She had been issued a medication review over the phone for the last 8 years. There was documentation 2 years ago that she was ‘aware of the risks’.*** | ***7*** | ***Probably***  ***Avoidable*** |
| 2 | She had been on combined continuous HRT for 5 years. She was a ‘social smoker’ and had annual face-to-face reviews with BMI measurements, smoking cessation advice. She’d been given written information as to the risks and benefits every year. She had agreed to face the risks of taking HRT – this was documented as an informed decision in the notes. | 2 | Probably  Unavoidable |
| 3 | ***She had been on combined continuous HRT for 2 months. She had no documented BMI (it was 38), no documentation of discussion of risks or benefits and had not been asked about family history of VTE (her sister had a DVT)*** | ***8*** | ***Probably***  ***Avoidable*** |
| 4 | She was commenced on a 3-month trial of HRT. She had no excess risk factors – and these were documented. She was given written information about risks and benefits. She was given time to go away and think about the benefits and risks. She made an informed choice to commence treatment. | 1 | Totally  Unavoidable |
| 5 | Patient is on HRT for 2 years. She is on it during hospital admission for a DVT. The junior doctor running the clinic does not recognize this fact as a potential cause. She is not advised to stop the HRT by the hospital team. In between she moves to a new house and registers with a new GP who is not aware of her DVT as treatment for it has stopped in the interim and no discharge letters have been sent. 2 weeks after stopping her Dabigatran, she is admitted with a saddle embolus. | 4 | Possibly  Avoidable |
| **R.** | **A man aged 55 has a stroke** |  |  |
| 1 | He had a BP reading 3 months before his stroke at his local pharmacy whilst buying nicotine replacement therapy, it was 180/115. There is no system for the pharmacy to inform the GP. He was advised to go and see his GP about his blood pressure, but no information about the urgency or why he might want to be offered. | 1 | Totally  Unavoidable |
| 2 | ***He saw the practice nurse for a ‘general checkup’ as his brother has recently had a stroke at the age of 60. The BP was 165/100 and he was advised to buy his own machine, take some readings and see the GP. He has a low paid job and cannot afford to miss work to see GP or to buy a machine. Bloods taken on the day show a Total Cholesterol:HDL ratio of 7 and CVS 10-year risk 35%. There is no system in place in the practice to recall patients with high cholesterol and as he hasn’t seen a GP, no one pays attention to his result.*** | ***8*** | ***Probably***  ***Avoidable*** |
| 3 | The man has never seen a doctor. He wakes suddenly one morning with a dense right-sided weakness, despite being a non-smoker and having no risk factors for stroke. | 1 | Totally  Unavoidable |
| 4 | He was admitted to hospital for an elective hernia repair. He was found to have a BP of 180/90 which was controlled peri-operatively. No mention of this is made in the discharge letter. The patient is not called for review and wasn’t told to see his GP. He had a stroke 2 months later. BP on arrival was 210/90. | 1 | Totally  Unavoidable |
| 5 | He was seen complaining of a fleeting sensation of tingling in his left ring finger 2 days previously. Nil is found on his presentation at GP, with no obvious risk factors for Stroke identified. 3 days later he had a stroke with permanent weakness of his left arm. Carotid Doppler show 90% occlusion of his right carotid. | 2 | Probably  Unavoidable  Without  Consensus |
| **S.** | **A man aged 35 with Schizophrenia taking Olanzapine presents with a collapse due to prolonged QT syndrome** | | |
| 1 | The CMHT ask the GP to monitor olanzapine as per the local shared care agreement. They do not send the agreed documentation and so the GP surgery refuse to take over care and do an ECG. The ECG is never done due to a refusal by both sides to complete appropriate paperwork and prolonged QT is missed. | 7 | Probably Avoidable Without Consensus |
| 2 | He takes Olanzapine for 3 months before the GP receives a letter requesting that they carry out an ECG. The GP doesn’t feel he is qualified to fully interpret any ECG and so refers to cardiology, in the meantime, patient is admitted. | 5 | Possibly  Avoidable  Without  Consensus |
| 3 | ***A GP sees an ECG from the patient as part of an agreed shared care arrangement of this patient and misreads the ECG as normal. The ECG on review, shows a prolonged QT interval.*** | ***8*** | ***Probably***  ***Avoidable*** |
| 4 | The patient arranges an annual review with his usual GP with whom he has a good relationship , including an ECG the day before with the nurse, at 14 months rather than the recommended 12 months since the GP is on annual leave. The day before the appointment with the nurse, he collapses. | 2 | Probably  Unavoidable |
| 5 | The patient complies with all monitoring of his Olanzapine. He has an episode of collapse (requiring admission) due to taking a drug with an interaction causing an arrhythmia. The patient was told about this interaction, and given written information. The patient forgot about this particular drug and is unable to read. | 3 | Probably  Unavoidable  Without  Consensus |
| **T.** | **A woman aged 50 is diagnosed with Bladder Cancer** | | |
| 1 | ***The patient had presented with symptoms of frequency and pain and despite not having any bacterial growth on several MSU, but had red blood cells 5-99 on everyone. The GP seeing her did not realize the relevance of this finding and referred her as a routine referral and not less than 2 weeks wait. She had metastatic disease after an 18 week wait*** | ***8*** | ***Probably***  ***Avoidable*** |
| 2 | ***The patient had red blood cells on a urine dipstick on a new patient medical with the nurse. This is documented, but not followed up on as the practice has no system to allow for this. 6 months later, she presents with frank hematuria. She had invasive disease and requires radical surgery.*** | ***8*** | ***Probably***  ***Avoidable*** |
| 3 | On a routine medical examination, she is found to have hematuria (microscopic). She is referred urgently to urology under 2-week wait. She is diagnosed with cancer. | 1 | Totally  Unavoidable |
| 4 | A patient is found to have microscopic hematuria. She is referred to a urologist. She has a cystoscopy. The histology of a lesion is found to be CIS and follow up is arranged. The hospital records have the wrong address and so the follow up letter goes to the wrong house. By the time she has been re-referred, it is 6 months later and the cancer is a T3 cancer and requires surgery. (Unavoidable Without Consensus) | 3 | Probably  Unavoidable  Without  Consensus |
| 5 | ***The patient has macroscopic hematuria and is treated for a urine infection. The blood macroscopically clears but no follow up is arranged. 6 months later she has another episode and this time is referred urgently as she has a family history of bladder cancer. She is found to have T4 cancer.*** | ***7*** | ***Probably***  ***Avoidable*** |
